# Complications during CT-Guided Lung Nodule Localization: Impact of Needle Insertion Depth and Patient Characteristics

**DOI:** 10.3390/diagnostics13111881

**Published:** 2023-05-27

**Authors:** Hua Chiang, Liang-Kuang Chen, Wen-Pei Hsieh, Yun-Xuan Tang, Chun-Yu Lo

**Affiliations:** 1 Department of Diagnostic Radiology, Shin Kong Wu Ho-Su Memorial Hospital, Taipei 11101, Taiwan; 2 Department of Medical Imaging and Radiological Technology, Yuanpei University of Medical Technology, Hsinchu 30015, Taiwan

**Keywords:** computed tomography-guided localization, complications, pulmonary hemorrhage, pneumothorax, sex, lung lobe

## Abstract

Although widely used, CT-guided lung nodule localization is associated with a significant risk of complications, including pneumothorax and pulmonary hemorrhage. This study identified potential risk factors affecting the complications associated with CT-guided lung nodule localization. Data from patients with lung nodules who underwent preoperative CT-guided localization with patent blue vital (PBV) dye at Shin Kong Wu Ho-Su Memorial Hospital, Taiwan, were retrospectively collected. Logistic regression analysis, the chi-square test, and the Mann–Whitney test were used to analyze the potential risk factors for procedure-related complications. We included 101 patients with a single nodule (49 with pneumothorax and 28 with pulmonary hemorrhage). The results revealed that men were more susceptible to pneumothorax during CT-guided localization (odds ratio: 2.48, *p* = 0.04). Both deeper needle insertion depth (odds ratio: 1.84, *p* = 0.02) and nodules localized in the left lung lobe (odds ratio: 4.19, *p* = 0.03) were associated with an increased risk of pulmonary hemorrhage during CT-guided localization. In conclusion, for patients with a single nodule, considering the needle insertion depth and patient characteristics during CT-guided localization procedures is probably important for reducing the risk of complications.

## 1. Introduction

Computed tomography (CT)-guided procedures, including lung nodule localization and biopsy, are safe and widely used but are associated with a high risk of complications [1,2,3,4,5,6,7], the most common being pneumothorax and pulmonary hemorrhage [2,3,4,7]. Although most cases of pneumothorax and pulmonary hemorrhage are asymptomatic and self-resolving, a small proportion of cases require chest tube drainage [3,4,7]. Many studies on CT-guided localization have demonstrated that a considerable proportion of patients develop iatrogenic pneumothorax and pulmonary hemorrhage [2,8,9]. For instance, Lin et al.’s research reported that iatrogenic pneumothorax occurred in 54.2% of cases, while pulmonary hemorrhage was observed in 29.4% of cases [2]. Moreover, these complications might result in delayed surgery and affect subsequent treatment plans. Occasionally, the complications can be life-threatening, leading to immense psychological pressure on physicians and potential medical disputes. Thus, factors influencing these complications need to be characterized.

Most studies on CT-guided localization have primarily focused on the assessment of procedural safety and efficacy [2,8,9,10,11,12,13,14,15,16,17]. By contrast, the discussion of the factors influencing complications resulting from CT-guided localization is frequently secondary [9,11,12,15]. Few studies have specifically examined the factors influencing the complications associated with CT-guided localization [18]. Numerous factors might possibly influence the occurrence of complications during CT-guided localization, including nodule size, nodule depth, needle insertion depth, age, underlying chronic diseases, sex, lung lobes involved, imaging results, patient position during the procedure, the type and size of the needle employed, operator experience (years of experience), the type of scanner employed for guiding the procedure, and the need to cross the fissure. A thorough investigation of these factors can fill the literature gap and help clinicians avoid the aforementioned risks.

In this study, we explored the potential factors affecting the occurrence of complications during CT-guided lung nodule localization.

## 2. Materials and Methods

### 2.1. Participant Source

Between August 2014 and March 2021, 155 patients with incidentally detected lung nodules underwent preoperative CT-guided localization with PBV dye followed by video-assisted thoracoscopic resection at Shin Kong Wu Ho-Su Memorial Hospital, Taiwan. We retrospectively retrieved the following data from the patients’ electronic medical records: nodule size, nodule depth, needle insertion depth, age, preexisting comorbidities (diabetes mellitus, hypertension, and hyperlipidemia), sex, and affected lung lobes. This study was approved by the Research Ethics Committee of Shin Kong Wu Ho-Su Memorial Hospital (no. 20221006R).

### 2.2. Methods for CT-Guided Localization with PBV Dye

The preoperative CT images of all patients were reviewed by at least two radiologists to determine whether preoperative lung nodule localization was required [19,20]. Before the procedure, the patients and their families were informed about the risks and potential complications, including pneumothorax and pulmonary hemorrhage. Experienced radiologists, radiographers, and nurses performed the localization procedures using a 64-slice CT scanner (SOMATOM Definition AS, Siemens Healthineers, Forchheim, Germany) with a low-dose, thin-slice protocol (parameters: 1-mm thickness, 0.6 pitch, 0.5 s/rotation, 100 kV, and 40 mA).

**Figure 1:**
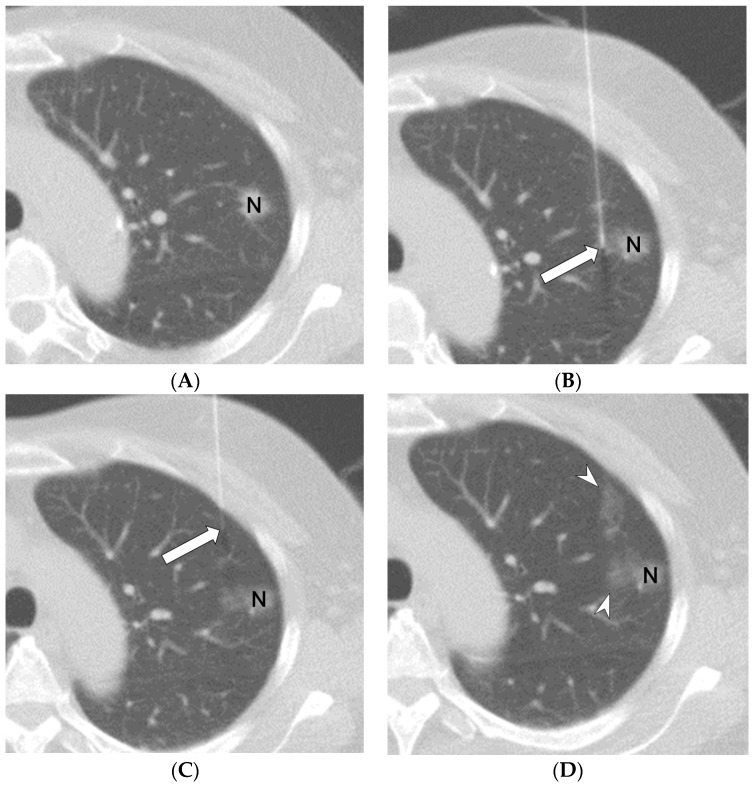
A 65-year-old woman undergoing CT-guided localization with PBV dye. Preprocedural chest CT revealed a 1.0-cm part-solid nodule (N) in the left upper lobe ((**A**), CT axial view). Two PBV dye doses were then slowly injected, with the first localization site being placed near the deepest point of the target lesion (N) in the lung parenchyma ((**B**), white arrow) and the second site near the pleural surface of needle entry ((**C**), white arrow). PBV dye was then administered, resulting in the nodule (N) being clearly marked ((**D**), white arrowheads).

The procedure was based on relevant studies and was performed in the hospital’s CT room [2,13]. The patient was positioned in the prone, supine, or lateral decubitus position, and the lesion side was prepared for optimal needle path planning (CTs, Figure 1A). All patients were administered the same anesthetic (2% lidocaine HCl, Tai Yu, Taiwan). Under CT guidance, a 22-gauge Chiba needle was inserted toward the lesion until the needle tip reached it. Two doses of PBV dye (2.5%; Guerbet, Aulnay-sous-Bois, France) were slowly injected: the first localization site was near the deepest point of the target lesion in the lung parenchyma (Figure 1B), and the second was near the pleural surface of needle entry (Figure 1C). The total PBV dye volume was 0.2–0.3 mL (0.1–0.2 mL per area), and the nodules were easily identifiable during excision (Figure 1D).

A final CT image was taken before procedure completion to ensure the complete coverage of the nodules with the dye and to screen for any life-threatening complications such as pneumothorax or pulmonary hemorrhage. After the procedure, preparations for surgery were made.

### 2.3. Experimental Design and Methods

Patients with and without complications were considered the case and control groups, respectively, and the potential factors affecting complications were compared between the groups.

First, we analyzed the relationship between the complications of pneumothorax pulmonary hemorrhage with various potential factors. Next, these risk factors were compared between the case and control groups through pairwise comparisons to identify significant differences. Finally, for numerical risk factors, we attempted to establish a threshold to predict the occurrence of complications during CT-guided localization.

We assigned values of 1 for patients with pneumothorax or pulmonary hemorrhage and 0 for those without. Continuous variables included nodule size (cm), nodule depth (cm), needle insertion depth (cm), patient age (years), and operator experience (years). Categorical variables included diabetes mellitus, hypertension, hyperlipidemia, sex, lung localization, and the need to cross the fissure; the presence of comorbidities, male sex, left lung, and the need to cross the fissure was indicated as 1, and the absence of comorbidities, female sex, right lung, or no need to cross the fissure was indicated as 0.

Furthermore, subcategorized lung lobes, including LLL (left lower lobe), LUL (left upper lobe), RLL (right lower lobe), RML (right middle lobe), and RUL (right upper lobe), imaging results such as GGN (ground glass nodule), PSN (part-solid nodule), and SN (solid nodule), and patient position during the procedure, including supine, prone, left decubitus, and right decubitus, were also discussed. The target variables were assigned a value of 1, and the other categories were assigned a value of 0. For example, when analyzing the LLL, it was indicated as 1, while LUL, RLL, RML, and RUL were set to 0, representing non-LLL.

Finally, as we used the same type of needle (22-gauge Chiba needle) and the same type of scanner, a 64-slice CT scanner (SOMATOM Definition AS, Siemens Healthineers, Forchheim, Germany), for guiding the procedure in all cases, these factors were not included in the statistical analysis.

### 2.4. Definition of General Data

All nodules were evaluated using certain lung window settings (window level: −500 HU, window width: 1500 HU) and were imaged in sagittal, axial, and coronal views. We collected the patients’ basic clinicodemographic data from their electronic medical records and their CT images, including the presence of pneumothorax, presence of pulmonary hemorrhage, number of nodules, age, operator experience, diabetes mellitus, hypertension, hyperlipidemia, sex, location of nodules, date of examination, imaging results, patient position during the procedure, procedure across the fissure, and pathology results.

The grade of pneumothorax was classified based on the 2010 British Thoracic Society guideline [21]. A “large” pneumothorax was distinguished from a “small” one by the presence of a visible gap greater than 2 cm between the lung margin and the chest wall at the level of the hilum. The intensity of pulmonary hemorrhage was assessed using a modified grading system proposed by Tai et al. [22]. Grade 1 was defined as needle tract hemorrhage 2 cm or less in width, grade 2 as hemorrhage more than 2 cm in width but sublobar, grade 3 as lobar hemorrhage or greater, and grade 4 as hemothorax.

Data on the clinical characteristics of the nodules were obtained from the final preoperative CT images. Nodule size was defined as the maximum diameter of the nodule, nodule depth was estimated as the distance from the nodule margin to the nearest pleura, and needle insertion depth was defined as the needle length inside the pleura. All data represent the most appropriate lengths or distances selected across all CT slices. Patient age (years) and operator experience (years) are primarily adjusted and calculated based on the baseline year at the time.

Diabetes mellitus was diagnosed as the presence of any of the following criteria: HbA1C > 6.5%, fasting plasma glucose >126 mg/dL, or plasma glucose ≥200 mg/dL during a 2-h oral glucose tolerance test [23]. Hypertension was defined as blood pressure >140/90 mmHg [24]. Hyperlipidemia was defined as the presence of any of the following criteria: (a) total cholesterol ≥220 mg/dL, (b) LDL cholesterol ≥140 mg/dL, (c) HDL cholesterol <40 mg/dL, or (d) triglycerides ≥150 mg/dL [25]. Sex differences were primarily based on the physiological definitions of men and women and did not include intersex individuals [26]. The left and right lungs were defined based on their anatomical structures [27].

### 2.5. Inclusion and Exclusion Criteria

Because complication rates differ between multiple and single nodules and because most patients in our cohort had a single nodule, we used single nodules as the standard for analysis [9,12]. The inclusion criteria were based on those used in previous studies based on the research of Kong et al. assessing individuals with a single lung nodule [11]: (a) being 18–80 years old; (b) having a single small lung nodule measuring 5–15 mm in diameter that was diagnosed by at least two radiologists as being potentially malignant; (c) having no imaging evidence of emphysema, pulmonary hypertension, or pulmonary fibrosis; (d) having intraoperative and postoperative pathological results; (e) having no obvious vasculitis, bullae, or other important tissues or structures in the needle puncture path; and (f) having normal coagulation function. We excluded patients with (a) distant metastases on images; (b) severe heart, liver, lung, or brain disease or severe infection within 1 month prior to CT-guided localization with PBV dye; (c) imaging evidence of severe pleural effusion or lung injury; (d) two or more lung nodules on imaging; or (e) two or more punctures during CT-guided localization.

### 2.6. Statistical Analysis

First, a univariate logistic regression analysis was conducted, and significant variables were included in a multivariate binary logistic analysis [11,28,29]. Categorical variables were analyzed using the chi-square test, and non-normally distributed continuous variables were analyzed using the Mann–Whitney test. Categorical variables are presented as numbers and percentages; numerical variables are presented as medians and interquartile range (IQR) or means and standard deviation (SD). Receiver operating characteristic (ROC) curves were used to determine the optimal cutoff values for predicting complications during CT-guided localization.

The resulting data were rounded up to two decimal places unless the *p* value is between 0.001 and 0.01. A two-tailed *p* value of <0.05 was set as statistically significant. All descriptive statistics were calculated with MedCalc statistical software version 20.216 (MedCalc Software, Ostend, Belgium).

## 3. Results

### 3.1. Screened and Included Patient Data

Of the initial 155 patients sampled, 101 (34 men and 67 women) were eligible for this study (Table 1). Among them, 39 patients had no complications, 62 experienced at least one complication, 49 suffered from pneumothorax, 28 encountered pulmonary hemorrhage, and 15 had both pneumothorax and pulmonary hemorrhage. All patients who experienced pneumothorax had small and asymptomatic conditions, requiring only oxygen administration through a nasal cannula, with no instances of large pneumothorax. Furthermore, among the patients who experienced pulmonary hemorrhage, 26 encountered grade 1 and 2 experienced grade 2, with no cases of grade 3 or 4 pulmonary hemorrhage. All these patients remained asymptomatic.

The Kolmogorov–Smirnov test revealed that the patient data for nodule size, nodule depth, needle insertion depth, and operator experience did not follow a normal distribution, whereas the patient data for age conformed to a normal distribution. The median nodule size was 0.91 cm (IQR: 0.70–1.20), the median nodule depth was 0.67 cm (IQR: 0.38–1.52), the median needle insertion depth was 1.75 cm (IQR: 1.18–2.87), and the median operator experience was 5 years (IQR: 2–11). The mean patient age was 57.75 ± 11.11 years.

Furthermore, 21 patients had diabetes mellitus, 38 had hypertension, and 39 had hyperlipidemia. The procedure was performed on 48 left lung lobes, with 21 LLL and 27 LUL, as well as 53 right lung lobes, including 27 RLL, 6 RML, and 20 RUL. Nodules included 60 ground-glass nodules, 24 part-solid nodules, and 17 pure solid nodules. Patient positions during the procedure were 41 supine, 44 prone, 6 left decubitus, and 10 right decubitus. 4 procedures required crossing the fissure. Pathological diagnoses comprised primary adenocarcinoma (n = 71), atypical adenomatous hyperplasia (n = 5), hamartoma (n = 4), squamous cell carcinoma (n = 3), and benign nodules (n = 18), with various benign subtypes.

### 3.2. Potential Factors and Pneumothorax Caused by CT-Guided Localization

The univariate logistic regression analysis indicated that only the needle insertion depth (*p* = 0.04) and sex (*p* = 0.02) were the predictors of pneumothorax during CT-guided localization; however, the multivariate regression analysis indicated that only sex (*p* = 0.04) was the significant predictor (Table 2). Furthermore, men were more likely to experience pneumothorax during localization than women (odds ratio: 2.48, Table 2).

### 3.3. Potential Factors and Pulmonary Hemorrhage Caused by CT-Guided Localization

The univariate logistic regression analysis revealed that only the nodule depth (*p* = 0.003), needle insertion depth (*p* < 0.001), affected lung lobe (*p* = 0.001) and left upper lobe (*p* =0.002) were predictors of pulmonary hemorrhage during CT-guided localization. Nevertheless, the multivariate regression analysis results indicated that only the needle insertion depth (*p* = 0.02) and the lung lobe undergoing localization (*p* = 0.03) were significant predictors of iatrogenic pulmonary hemorrhage (Table 3). Moreover, deeper needle insertion and left lung procedures increased the risk of pulmonary hemorrhage (odds ratios: 1.84 and 4.19, respectively, Table 3).

### 3.4. Factors Associated with Pneumothorax and Pulmonary Hemorrhage Caused by CT-Guided Localization

Pairwise comparisons revealed that a significantly higher proportion of men (64.7%, n = 22) than women (40.3%, n = 27) experienced iatrogenic pneumothorax (*p* = 0.02, Table 4). The needle insertion depth (non-normally distributed) in patients with pulmonary hemorrhage (median: 2.70 cm, interquartile range [IQR]: 1.54–3.81 cm, n = 28) was longer than that in those without hemorrhage (median: 1.50 cm, IQR: 1.10–2.41 cm, n = 73, *p* < 0.001; Table 4). Finally, there was a significantly higher rate of CT-guided localization-induced pulmonary hemorrhage in the left lung (n = 21, 43.7%) than in the right lung (n = 7, 13.2%, *p* < 0.001; Table 4).

### 3.5. ROC Curve Analysis of Needle Insertion Depth

**Figure 2:**
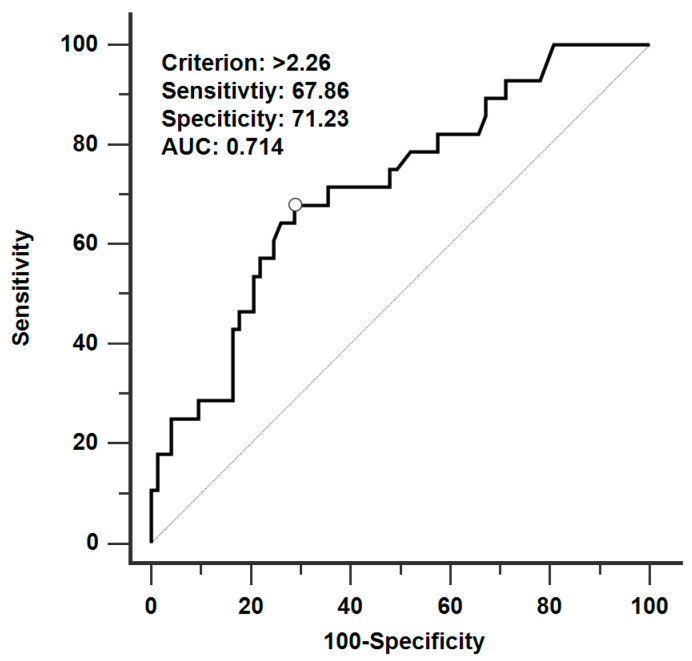
ROC curve analysis results of needle insertion length and pulmonary hemorrhage during CT-guided localization with PBV dye. The sample size is 101, with an area under the curve (AUC) of 0.714 and a *p* < 0.001. The optimal threshold for predicting pulmonary hemorrhage was a needle insertion depth greater than 2.26 cm, with a sensitivity of 67.86% and a specificity of 71.23%.

The ROC analysis results demonstrated that although the needle insertion depth was not highly accurate, it could be used to predict the occurrence of iatrogenic pulmonary hemorrhage (area under the curve: 0.714, *p* < 0.001, Figure 2), and the optimal threshold for predicting pulmonary hemorrhage incidence during CT-guided localization was a needle insertion depth of >2.26 cm (sensitivity: 67.86%, specificity: 71.23%; Figure 2).

## 4. Discussion

This study explored the influence of patient characteristics on the incidence of pneumothorax and pulmonary hemorrhage during CT-guided lung nodule localization. Our results revealed that men had a significantly higher risk of pneumothorax (*p* = 0.04) and that deeper needle insertion (*p* = 0.02) and left lung nodules (*p* = 0.03) significantly increased the likelihood of pulmonary hemorrhage. Thus, clinicians should pay more attention to these parameters when assessing the suitability of performing CT-guided lung nodule localization in patients with a single nodule, thereby reducing the risk of iatrogenic complications.

Studies have reported that men have a much higher incidence of spontaneous pneumothorax than women [30,31]. A study also reported a higher likelihood of iatrogenic pneumothorax in men during CT-guided localization [18]. Our results are consistent with these previous findings; however, we excluded patients with multiple nodules to reduce the potential interference from the simultaneous analysis of single and multiple nodule cases. This approach might address the differing complication rates for single and multiple nodules in previous studies that may have influenced multivariate regression analysis outcomes [9,12]. Furthermore, our study consistently used PBV dye for localization, whereas a previous study used various methods, such as hook wires, for deeper nodules [18]. Our approach therefore has the advantage of a unified complication rate; moreover, the rates of pneumothorax and pulmonary hemorrhage are higher with hook wires [2].

Contrary to our findings and those of Huang et al., Lin et al. suggested that sex is not a risk factor for pneumothorax [12]. In the study by Lin et al., sex was also found to have a significant difference in univariate regression analysis. In their research, procedure time was identified as an influencing factor for pneumothorax, and they also incorporated multiple localizations and the number of pleural punctures into their multivariate regression analysis. The aforementioned factors were not recorded and analyzed in our study, and their cases of iatrogenic pneumothorax included patients with multiple nodules. Our study also considered needle depth as a factor in the multivariate regression analysis and primarily focused on a more homogeneous patient population for analysis, such as those with a single nodule, single needle puncture, or no distant metastasis on imaging. The differences in patient populations and analyzed factors may contribute to the variations in the analysis results. In research regarding CT-guided biopsy procedures, which typically use thicker, true-cut needles, the relationship with pneumothorax does not seem to be affected by gender. For instance, studies by Drumm and Hiraki found that gender does not influence the incidence of pneumothorax in patients [32,33]. However, our findings suggest an intriguing phenomenon: thinner needles may increase the likelihood of pneumothorax in males. In the future, it might be worth exploring whether different needle sizes could influence the effect of gender on pneumothorax. Although the aforementioned previous studies have provided consistent and inconsistent conclusions [12,18], our results apply specifically to patients with a single nodule.

**Figure 3:**
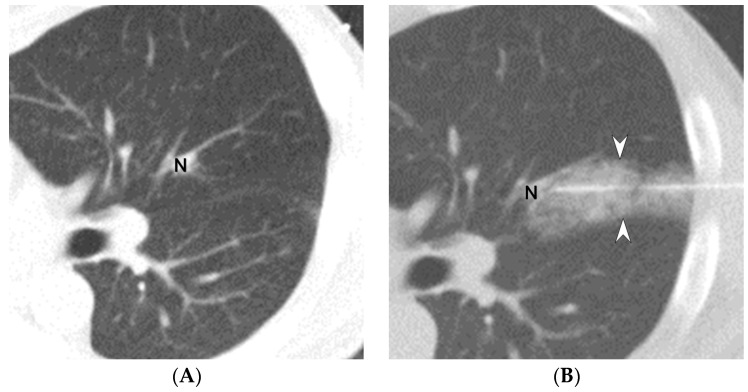

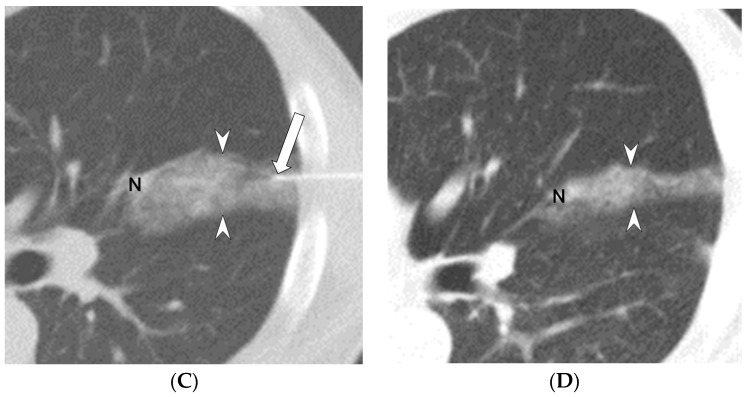
A 49-year-old man undergoing CT-guided localization with PBV dye. Preprocedural chest CT revealed a 0.87-cm part-solid nodule (N) in the left upper lobe ((**A**), CT axial view). Following a 4.80-cm needle insertion localization, CT images indicated that consolidation and the ground-glass opacities ((**B**), white arrowheads) to the left side of the nodule (N), and these findings were consistent with grade 2 pulmonary hemorrhage (**B**). The needle was then slowly relocated closer to the pleural area ((**C**), white arrow). A significant amount of pulmonary hemorrhage was observed within the patient’s body ((**D**), white arrowheads).

Our findings indicate that deeper needle insertion is associated with an increased risk of pulmonary hemorrhage during CT-guided localization (CTs, Figure 3A–D). Moreover, we conducted an ROC analysis to determine the optimal cut-off value (>2.26 cm) for predicting pulmonary hemorrhage during CT-guided localization. However, we have also noticed that our needle insertion length appears to be shorter compared to other studies (In our study, the median needle insertion depth was 1.75 cm, whereas in Lin’s study the mean was 1.83 cm and in Tsai’s study the median was 3.6 cm) [2,16]. We believe this discrepancy might be due to the fact that the patients included in our study had only a single nodule and were not complex cases. Lin’s study included patients with multiple nodules, and Tsai’s study primarily discussed patients with deeply situated nodules [2,16]. Indeed, the patient conditions included in our study may result in a shorter needle insertion length. A study reported that nodule depth affects the incidence of iatrogenic pulmonary hemorrhage [12]. However, based on the univariate analysis results, the needle insertion depth was not included in the multivariate regression analysis [12]. Although the relationship between the nodule depth and needle insertion depth remains unclear, the nodule depth probably affects the needle length and path taken by the physician during localization. Our study included both the nodule depth and needle insertion depth in the multivariate regression analysis, enabling the comparison of their prediction abilities for the incidence of iatrogenic pulmonary hemorrhage. Although the relationship of the needle insertion depth with pulmonary hemorrhage during CT-guided biopsy has been discussed [3,4], our findings supplement the knowledge of the impact of the same risk factor in CT-guided localization.

Our results also revealed that the risk of iatrogenic pulmonary hemorrhage was higher during CT-guided localization in the left lung. Based on our literature review, we might be the first study to discover that pulmonary hemorrhage caused by CT-guided localization is more likely to occur in the left lung. Although the literature review did not allow any related conclusions, it can be hypothesised that the larger air volume of the right lung compared to the left lung led to more available imaging-guided needle space that can be adjusted to avoid close proximity to potential vessels on the images [34]. Lin et al. concluded that lung lobes (e.g., left lower lobe and right upper lobe) were not associated with the incidence of pulmonary hemorrhage during CT-guided localization [12]. Unlike their study, we analyzed not only left and right lung lobes but also more subcategorized lung lobes in the present study. Additionally, the proportion of left and right lung lobes was nearly equal in our analysis, with right lung lobes comprising 52.48% and left lung lobes accounting for 47.58%, and only single nodules were included in this study, likely increasing the accuracy of our multivariate regression analysis results. Moreover, we did not use any specific subcategorized lung lobe as a reference; instead, we individually analyzed each subcategorized lung lobe in comparison to the others. However, we also found no significant difference in the conclusions regarding subcategorized lung and iatrogenic pulmonary hemorrhage; the only difference was observed between the left and right lung lobes. In studies on CT-guided biopsy and pulmonary hemorrhage, no particular effect of lung lobes on pulmonary hemorrhage has been noted. For example, Zhu’s research, which employed similar multivariate analysis, also found no significant differences [35]. We believe this may be related to the thickness of the needle, but more research is needed to support this hypothesis. In a previous study and our conclusion, no significant difference was observed in pulmonary hemorrhage during CT-guided localization between more subcategorized lung lobes [12]. However, according to the conclusions of this study, clinicians should probably exercise caution when performing CT-guided localization in patients with left lung nodules.

In our study, operator experience (years) did not affect the occurrence of complications. This could be due to the fact that while seniority may increase operational experience, different operators may have varying aptitudes and mastery levels for CT-guided localization. Similar matters were discussed in the study by Huang, but no significant differences in pneumothorax were observed among the three operators [18]. Additionally, the condition of the operator on the day of the procedure and the level of patient cooperation might also play a role, which is another topic worth exploring in the future.

This study has several limitations. First, we excluded patients with multiple nodules or distant metastases [11], thus precluding the generalizability of our results to those patient groups. Second, some potential factors that were not analyzed may also influence the incidence of complications, such as the procedure time and puncture frequency [12,18]. Third, this study had the strength of having multiple attending physicians interpret the images. However, this may introduce human error due to variations among the execution teams (eleven teams performed the CT-guided localization procedures). Ensuring the same team composition may increase the accuracy of the results, as observed in previous studies [16]. Fourth, this study did not incorporate patients who required intubation due to pneumothorax or pulmonary hemorrhage, decreased blood oxygen levels, or alterations in vital signs during CT-guided localization in the analysis. This raises concerns about applying the study results to patients with severe iatrogenic pneumothorax and pulmonary hemorrhage. Including patients with severe pneumothorax or pulmonary hemorrhage during CT-guided localization may help address this gap. Finally, this was a retrospective, nonrandomized cohort study focusing solely on CT-guided localization; our findings cannot be extrapolated to other procedures such as biopsy, radiofrequency treatment, or microwave ablation. Future studies might analyze data on multiple nodules or distant metastasis, consider additional potential risk factors, better control human factors such as having the same team perform the procedure, and include patients requiring intubation, having drops in blood oxygen levels, or having changes in vital signs during CT-guided localization to validate our findings.

## 5. Conclusions

Our data indicate that patients with a single nodule, male sex, deeper needle insertion, and a left lung may have an increased risk of complications during CT-guided localization procedures.

## Figures and Tables

**Table 1:** Patients’ clinicodemographic characteristics.

Variables	Number (Percentage)/Median (IQR) or Mean (SD)	Variables	Number (Percentage)
Patients	101 (100%)	**Patient position:**	
**Complications:**		Supine	41 (40.59%)
Pneumothorax	49 (48.51%)	Prone	44 (43.56%)
Pulmonary hemorrhage	28 (27.72%)	Left decubitus	6 (5.94%)
Nodule Size (cm): median (IQR)	0.91 (0.70–1.20) *	Right decubitus	10 (9.90%)
Nodule Depth (cm): median (IQR)	0.67 (0.38–1.52) *	**Need to cross the fissure**	4 (3.96%)
Needle Insertion Depth (cm): median (IQR)	1.75 (1.18–2.87) *	**Pathological results:**	
Age (years): mean ± SD	57.75 ± 11.11	Adenocarcinoma (N)	71 (70.30%)
Operator experience (years): median (IQR)	5 (2–11) *	Atypical adenomatous hyperplasia (N)	5 (4.95%)
**Underlying diseases:**		Hamartoma (N)	4 (3.96%)
Diabetes mellitus	21 (20.79%)	Squamous cell carcinoma (N)	3 (2.97%)
Hypertension	38 (37.62%)	Benign nodule (N)	18 (17.82%)
Hyperlipidemia	39 (38.61%)	Intrapulmonary lymph node (N)	4 (3.96%)
**Sex:**		Chronic inflammatory nodule (N)	3 (2.97%)
Male	34 (33.66%)	Granuloma (N)	2 (1.98%)
Female	67 (66.34%)	Organizing pneumonia nodule (N)	1 (0.99%)
**Lung lobe:**		Tuberculoma (N)	1 (0.99%)
Left lobe:	48 (47.52%)	Fibrotic nodule (N)	1 (0.99%)
Left lower lobe	21 (20.79%)	Minute pulmonary meningothelial-like nodule (N)	1 (0.99%)
Left upper lobe	27 (26.73%)	Chronic inflammatory and fibrotic nodule (N)	1 (0.99%)
Right lobe:	53 (52.48%)	Nodule with plasma cell infiltration (N)	1 (0.99%)
Right lower lobe	27 (26.73%)	Nodule with fungal infection (N)	1 (0.99%)
Right middle lobe	6 (5.94%)	Anthracofibrotic nodule (N)	1 (0.99%)
Right upper lobe	20 (19.80%)	Caseating granulomatous inflammatory nodule (N)	1 (0.99%)
**Imaging results:**			
Ground-glass nodule (N)	60 (59.40%)		
Part-solid nodule (N)	24 (23.76%)		
Solid nodule (N)	17 (16.83%)		

Categorical variables were presented as numbers and percentages. Numerical variables were presented as medians and interquartile range (IQR) or means and standard deviation (SD). * non-normally distributed.

**Table 2:** Potential factors affecting pneumothorax during CT-guided localization: univariate and multivariate analyses.

Variables	Univariate		Multivariate	
	OR (95%CI)	*p* Value	OR (95%CI)	*p* Value
Sex	2.72 (1.15–6.39)	*p* = 0.02 *	2.48 (1.03–5.94)	*p* = 0.04 *
Needle Insertion Depth	1.43 (1.02–2.01)	*p* = 0.04 *	1.38 (0.98–1.94)	*p* = 0.07
Nodule Size	1.73 (0.41–7.26)	*p* = 0.45		
Nodule Depth	0.92 (0.60–1.41)	*p* = 0.69		
Age	1.01 (0.98–1.05)	*p* = 0.44		
Operator Experience	1.00 (0.93–1.08)	*p* = 0.95		
Diabetes Mellitus	0.96 (0.37–2.50)	*p* = 0.93		
Hypertension	1.30 (0.58–2.92)	*p* = 0.52		
Hyperlipidemia	0.86 (0.38–1.91)	*p* = 0.71		
Lung Lobe	1.54 (0.70–3.38)	*p* = 0.28		
Left Lower Lobe	2.57 (0.94–7.05)	*p* = 0.07		
Left Upper Lobe	0.80 (0.33–1.94)	*p* = 0.62		
Right Lower Lobe	1.20 (0.50–2.90)	*p* = 0.69		
Right Middle Lobe	1.07 (0.20–5.55)	*p* = 0.94		
Right Upper Lobe	0.38 (0.13–1.08)	*p* = 0.07		
Ground Glass Nodule	0.60 (0.27–1.33)	*p* = 0.21		
Part-Solid Nodule	1.35 (0.54–3.38)	*p* = 0.53		
Solid Nodule	1.65 (0.57–4.74)	*p* = 0.35		
Supine	0.52 (0.23–1.17)	*p* = 0.12		
Prone	1.54 (0.70–3.39)	*p* = 0.29		
Left Decubitus	0.51 (0.09–2.92)	*p* = 0.45		
Right Decubitus	2.72 (0.66–11.19)	*p* = 0.17		
Need to cross the fissure	3.33 (0.33–33.11)	*p* = 0.31		

OR: odds ratio, 95% CI: 95% confidence interval. The data analysis used logistic regression. * *p* < 0.05.

**Table 3:** Potential factors affecting pulmonary hemorrhage during CT-guided localization: univariate and multivariate analyses.

Variables	Univariate		Multivariate	
	OR (95% CI)	*p* Value	OR (95% CI)	*p* Value
Needle Insertion Depth	1.91 (1.31–2.79)	*p* < 0.001 *	1.84 (1.09–3.09)	*p* = 0.02 *
Lung Lobe	5.11 (1.92–13.60)	*p* = 0.001 *	4.19 (1.11–15.77)	*p* = 0.03 *
Nodule Depth	2.12 (1.28–3.49)	*p* = 0.003 *	1.25 (0.61–2.55)	*p* = 0.54
Left Upper Lobe	4.62 (1.78–11.97)	*p* = 0.002 *	1.85 (0.51–6.69)	*p* = 0.35
Nodule Size	1.04 (0.21–5.12)	*p* = 0.96		
Age	1.02 (0.98–1.06)	*p* = 0.30		
Operator Experience	1.07 (0.98–1.16)	*p* = 0.12		
Diabetes Mellitus	1.40 (0.50–3.95)	*p* = 0.52		
Hypertension	0.89 (0.36–2.21)	*p* = 0.81		
Hyperlipidemia	0.54 (0.21–1.39)	*p* = 0.20		
Sex	1.13 (0.45–2.83)	*p* = 0.79		
Left Lower Lobe	1.40 (0.50–3.95)	*p* = 0.52		
Right Lower Lobe	0.36 (0.11–1.17)	*p* = 0.09		
Right Middle Lobe	0.50 (0.06–4.51)	*p* = 0.54		
Right Upper Lobe	0.24 (0.05–1.09)	*p* = 0.06		
Ground Glass Nodule	0.48 (0.20–1.16)	*p* = 0.10		
Part–Solid Nodule	1.83 (0.69–4.86)	*p* = 0.22		
Solid Nodule	1.54 (0.51–4.65)	*p* = 0.45		
Supine	1.14 (0.47–2.75)	*p* = 0.77		
Prone	0.64 (0.26–1.57)	*p* = 0.33		
Left Decubitus	0.50 (0.06–4.51)	*p* = 0.54		
Right Decubitus	2.96 (0.78–11.14)	*p* = 0.11		
Need to cross the fissure	2.73 (0.37–20.40)	*p* = 0.33		

OR: odds ratio, 95% CI: 95% confidence interval. The data analysis used logistic regression. * *p* < 0.05.

**Table 4:** Factors associated with pneumothorax and pulmonary hemorrhage during CT-guided localization.

Variables	Pneumothorax		
	With	Without	*p*-Value
Sex:			*p* = 0.02 ^1,^*
Men, n (%)	22 (64.7%)	12 (35.3%)	
Women, n (%)	27 (40.3%)	40 (59.7%)	
Variables	Pulmonary Hemorrhage		
	With	Without	*p*-Value
Needle Insertion Depth, median (IQR), cm Lung Lobe:	2.70 (IQR: 1.54–3.81, n = 28)	1.50 (IQR: 1.10–2.41, n = 73)	*p* < 0.001 ^2,^* p < 0.001 ^1,^*
Left Lung, n (%)	21 (43.7%)	27 (56.2%)	
Right Lung, n (%)	7 (13.2%)	46 (86.8%)	

Categorical variables are presented as counts and percentages, and numerical variables are presented as medians and IQR (interquartile range). ^1^ Categorical variables were analyzed using the Chi-Square test. ^2^ Numerical variables were analyzed with the Mann–Whitney test (non-normally distributed). * *p* < 0.05.

## Data Availability

Data is unavailable due to privacy or ethical restrictions.

## Abbreviations

CT = computed tomography, CTs = computed tomography, PBV = patent blue vital, LLL = left lower lobe, LUL = left upper lobe, RLL = right lower lobe, RML = right middle lobe, RUL = right upper lobe, GGN = ground glass nodule, PSN = part-solid nodule, SN = solid nodule, HbA1C = hemoglobin A1c, LDL = low-density lipoprotein, HDL = high-density lipoprotein, IQR = interquartile range, ROC = receiver operating characteristic.
